# Cdc42 defines apical identity and regulates epithelial morphogenesis by promoting apical recruitment of Par6-aPKC and Crumbs

**DOI:** 10.1242/dev.175497

**Published:** 2019-08-12

**Authors:** Francisca Nunes de Almeida, Rhian F. Walther, Mary T. Pressé, Evi Vlassaks, Franck Pichaud

**Affiliations:** 1 MRC - Laboratory for Molecular Cell Biology, University College London, London WC1E 6BT, UK; 2 Institute for the Physics of Living Systems, University College London, London WC1E 6BT, UK

**Keywords:** Epithelial polarity, Cdc42, Par6, Par3, Bazooka, aPKC, Crumbs, Par complex

## Abstract

Cdc42 regulates epithelial morphogenesis together with the Par complex (Baz/Par3-Par6-aPKC), Crumbs (Crb/CRB3) and Stardust (Sdt/PALS1). However, how these proteins work together and interact during epithelial morphogenesis is not well understood. To address this issue, we used the genetically amenable *Drosophila* pupal photoreceptor and follicular epithelium. We show that during epithelial morphogenesis active Cdc42 accumulates at the developing apical membrane and cell-cell contacts, independently of the Par complex and Crb. However, membrane localization of Baz, Par6-aPKC and Crb all depend on Cdc42. We find that although binding of Cdc42 to Par6 is not essential for the recruitment of Par6 and aPKC to the membrane, it is required for their apical localization and accumulation, which we find also depends on Par6 retention by Crb. In the pupal photoreceptor, membrane recruitment of Par6-aPKC also depends on Baz. Our work shows that Cdc42 is required for this recruitment and suggests that this factor promotes the handover of Par6-aPKC from Baz onto Crb. Altogether, we propose that Cdc42 drives morphogenesis by conferring apical identity, Par-complex assembly and apical accumulation of Crb.

## Abstract (precis)

**Summary:** During epithelia morphogenesis in *Drosophila*, the Rho-GTPase Cdc42 confers apical identity, Par-complex assembly and Crb accumulation to promote epithelial morphogenesis.

## INTRODUCTION

Epithelial cell polarity and morphogenesis involve the specification and maturation of distinct plasma membrane domains along the apical (top) to basal (bottom) axis of the cell. These domains support specific functions and consist of the apical membrane, which contains microvilli flanked by subapical membranes; the lateral membrane, containing junctional domains that mediate cell-cell adhesion and function as paracellular barriers; and the basal membrane. Polarized morphogenesis of these membrane domains depends on a set of proteins that are conserved through evolution. At the apical domain, these proteins include the small Rho-GTPase Cdc42 (Goldstein and Macara, 2007; Hoege and Hyman, 2013; Joberty et al., 2000; Johansson et al., 2000; Kemphues, 2000; Lin et al., 2000), the partitioning defective proteins Bazooka (Baz; Par3 in mammals) and Par6, the serine/threonine kinase aPKC (PKCζ/ι in mammals), the transmembrane protein Crb (CRB3 in mammals), and its binding partner Stardust (Sdt; PALS1 in mammals) (Flores-Benitez and Knust, 2016; Tepass, 2012).

*Drosophila* is a powerful model system for dissecting the mechanism of epithelial polarity and morphogenesis *in vivo*. In *Drosophila* cells such as the pupal photoreceptor and follicular epithelium, the zonula adherens (ZA), which contains E-cadherin (Ecad), marks the boundary between the apical and lateral membrane, and mediates cell-cell adhesion. Morphogenesis of the pupal photoreceptor requires the function of Cdc42, Baz, Par6, aPKC, Crb and Sdt (Hong et al., 2003; Izaddoost et al., 2002; Muschalik and Knust, 2011; Nam and Choi, 2003; Pellikka et al., 2002; Walther and Pichaud, 2010). In this sensory neuron, Baz is required for the recruitment of Par6, aPKC and Crb to the apical membrane (Hong et al., 2003; Walther et al., 2016; Walther and Pichaud, 2010). However, the converse is not true, as membrane recruitment of Baz towards the apical pole of the cell does not depend on Par6, aPKC and Crb (Walther et al., 2016; Walther and Pichaud, 2010). Similarly, in *baz*, *aPKC* and *crb* mutant cells, Ecad and Arm (β-catenin in *Drosophila*) are detected at the membrane, towards the apical pole of the cell. This suggests that apical localization of Baz and adherens junction material, such as Ecad and Arm, relies on cues other than those provided by Par6, aPKC and Crb. It also suggests that Baz and adherens junction material might act as apical cues during morphogenesis, and supports a model whereby Baz promotes the apical recruitment of Par6, aPKC and Crb. Similarly, Baz acts as an apical determinant in the cellularizing fly embryo. In this system, early apical localization of Baz does not depend on aPKC or Par6 (Harris and Peifer, 2005), and Baz is required for membrane localization of Par6-aPKC and Crb to support morphogenesis (Bilder et al., 2003; Harris and Tepass, 2008; Harris and Peifer, 2005; Harris and Peifer, 2007). The situation is somewhat different in the follicular epithelium, where Baz is dispensable for polarity, as well as for Par6, aPKC and Crb apical localization (Shahab et al., 2015). Altogether, this points to cell type-specific differences in the manner in which these proteins interact with each other to regulate polarity and morphogenesis.

An essential factor in epithelial polarity and morphogenesis is Cdc42 (Pichaud et al., 2019). In mammalian epithelial cells, it regulates lumen formation and junction maturation (Bryant et al., 2010; Jaffe et al., 2008; Pichaud et al., 2019). In the photoreceptor, Cdc42 is required for morphogenesis and membrane localization of Par6, aPKC and Crb (Walther and Pichaud, 2010). Cdc42 requirement for Baz, Par6 and aPKC membrane localization has also been shown in the remodelling fly neuroectoderm, where Cdc42 regulates the endocytosis of apical proteins through the Par complex (Harris and Tepass, 2008). In addition, Par6-aPKC membrane localization also depends on Cdc42 in the developing notum, where Cdc42 regulates the endocytosis of Ecad (Georgiou et al., 2008; Leibfried et al., 2008). Cdc42 requirement for Par6-aPKC membrane localization is linked to the ability of Cdc42 to bind to Par6 (Hutterer et al., 2004; Jin et al., 2015; Joberty et al., 2000). In the case of Cdc42 requirement for Crb membrane localization, it is likely to involve Par6-aPKC. This is the case in the pupal photoreceptor, where Cdc42 binding to Par6 is required for Crb apical accumulation (Walther and Pichaud, 2010). Similarly, in the neuroectoderm, Par6-aPKC mediates Cdc42 function in promoting Crb apical accumulation (Harris and Tepass, 2008). Furthermore, Par6 can be linked to Crb either through direct binding (Lemmers et al., 2004) or through Sdt/PALS1 (Hurd et al., 2003; Wang et al., 2004), and Par6 binding to Crb has been shown to be enhanced by Cdc42 (Kempkens et al., 2006; Lemmers et al., 2004; Whitney et al., 2016). However, how exactly Cdc42 activity and localization relates to that of the Par complex and Crb is not fully understood.

When considering how Cdc42, Baz, Par6-aPKC and Crb come together to regulate epithelial morphogenesis, an important step is the apical exclusion of Baz, which defines the apical membrane and ZA (Krahn et al., 2010; Morais-de-Sá et al., 2010; Walther and Pichaud, 2010). This exclusion results from the molecular sorting that is thought to occur when Crb outcompetes Baz binding to Par6 (Morais-de-Sá et al., 2010). During this step of molecular sorting, Par6 occupies an interesting position because it can also bind to Cdc42, aPKC and Sdt, which together with Baz and Crb, all contribute to promoting polarized morphogenesis (Fig. S1A). This makes Par6 an ideal candidate for coordinating Cdc42 activity, Par-complex assembly and Crb recruitment during epithelial morphogenesis. To test this possibility, we examined the relationship between Cdc42 localization and *baz*, *par6*, *aPKC* and *crb*. In addition, we performed an *in vivo* structure-function study of Par6 with a focus on disrupting its binding to Cdc42, aPKC and Crb. Altogether, our work in the photoreceptor and follicular epithelium shows that Cdc42 is active at the developing apical membrane and ZA*.* Our results indicate that apical localization of Par6-aPKC does not require Crb, and thus might rely on Par6 binding to Cdc42 or Baz. We also find that Cdc42 binding to Par6 is required for the stabilization and apical retention of Par6 by Crb. In turn, Par6 binding stabilizes Crb at the membrane and thus promotes Crb accumulation to support morphogenesis. Altogether, we propose that during epithelial morphogenesis Cdc42 is atop the protein network that determines the apical pole of the cell and promotes morphogenesis of the apical membrane and ZA.

## RESULTS

### Cdc42 defines apical identity

**Fig. 1.**
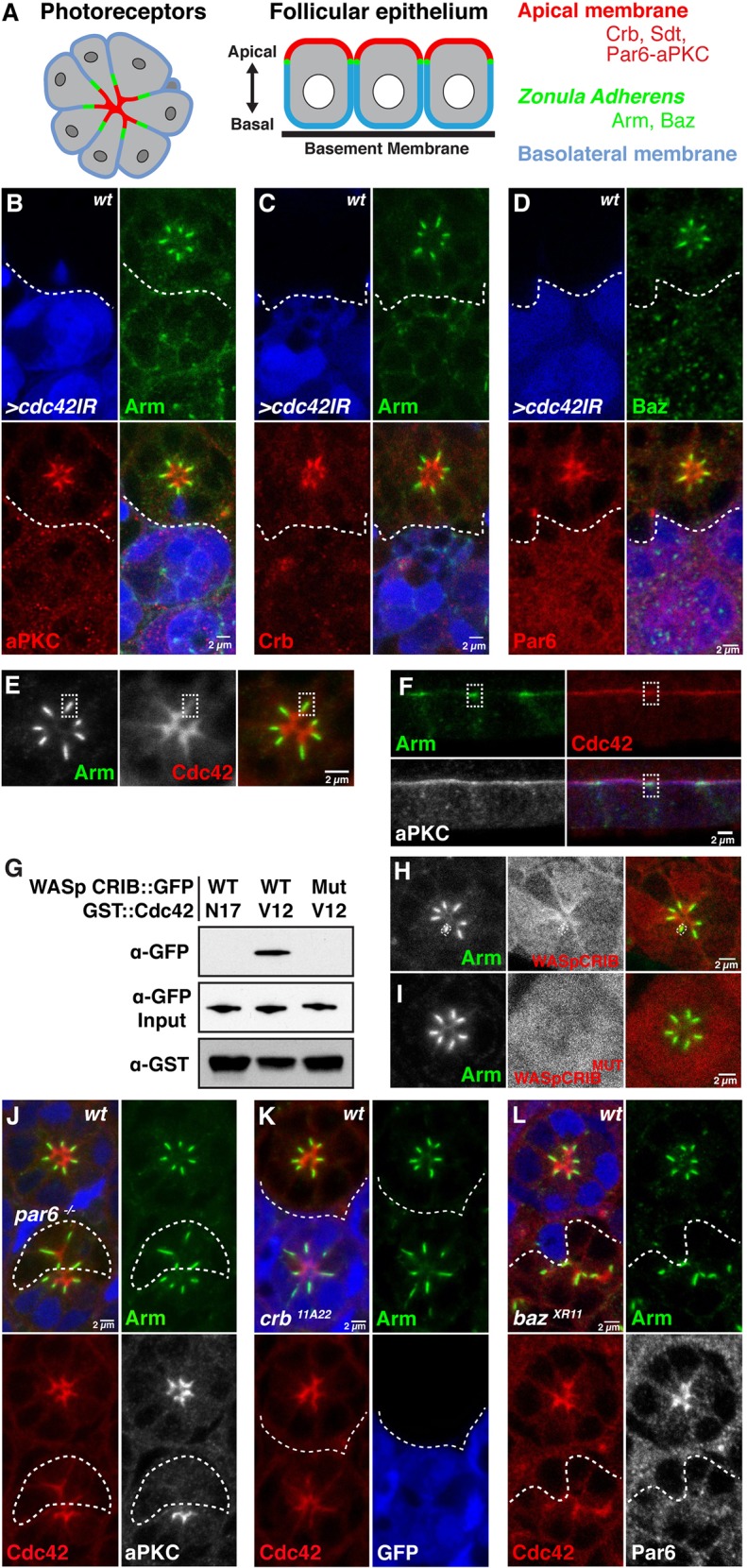
**Cdc42 defines apical identity.** (A) Schematic representation of developing fly photoreceptors at early-pupal stage and follicular epithelial cells. Photoreceptors are arranged in a circular cluster called an ommatidium. The apical membranes are in red, the ZA is in green and the basolateral membrane is in blue. (B-D) *Cdc42IR* marked by the presence of GFP (blue) and stained for Arm (B,C, green), aPKC (B, red), Crb (C, red), Baz (D, green) and Par6 (D, red). (E) mCherry::Cdc42 (red) and Arm (green) localization in the photoreceptor. (F) mCherry::Cdc42 (red), Arm (green) and aPKC (grey; blue in merged panel) localization in the follicular epithelium at stage 7. In E and F, the ZA is highlighted by a white dotted rectangle. (G) Representative pull-down experiment combining GST::Cdc42^V12^, GST::Cdc42^N17^ and the Cdc42-GTP probe WASp-CRIB::GFP or its mutated version (WASp-CRIB::GFP^MUT^) expressed in S2R+ cells. (H) Localization of Cdc42-GTP monitored using WASp-CRIB::GFP (red) and Arm (green). A white rectangle delineates a ZA to show that WASp-CRIB::GFP is detected in this membrane domain. (I) Control WASp-CRIB::GFP^MUT^ (red) and Arm (green). (J) *par6*^Δ*226*^ mutant cells labelled by the lack of GFP (blue), expressing mCherry::Cdc42 (red) and stained for Arm (green) and aPKC (grey). (K) *crb^11A22^* mutant cells positively labelled for GFP (blue), expressing mCherry::Cdc42 (red) and stained for Arm (green). (L) *baz^XR11^* mutant cells labelled by the lack of GFP (blue), expressing mCherry:: Cdc42 (red), and stained for Arm (green) and Par6 (grey). Scale bars: 2 µm.

In the early (37-40% APF) pupal photoreceptor (Fig. 1A), the apical membrane consists of poorly differentiated ruffles and is not yet subdivided into apical microvilli and subapical membrane (stalk) (Pichaud, 2018). Similarly, in the follicular epithelium, the apical membrane consists of microvilli with a very short subapical membrane and ZA (Tanentzapf et al., 2000) (Fig. 1A). In both cell types, Crb, Sdt and Par6-aPKC are enriched at the apical membrane, while Baz and Arm are enriched at the ZA (Fig. S1B-H) (Abdelilah-Seyfried et al., 2003; Franz and Riechmann, 2010; Hong et al., 2003; Izaddoost et al., 2002; Morais-de-Sá et al., 2010; Muschalik and Knust, 2011; Nam and Choi, 2003; Pellikka et al., 2002; Walther and Pichaud, 2010).

As the photoreceptor undergoes morphogenesis, Cdc42 is required for the apical recruitment of Par6-aPKC, Crb, Baz and Arm (Fig. 1B-D) (Walther and Pichaud, 2010). To assess where Cdc42 is localized in the photoreceptor, we made use of a functional mCherry::Cdc42 fusion protein (Abreu-Blanco et al., 2014). mCherry::Cdc42 expressed in *cdc42^3^* and *cdc42^4^* mutants rescued embryonic lethality and supported viability of adult animals. We found that mCherry::Cdc42 accumulates at the apical membrane and is also present at the photoreceptor ZA (Fig. 1E). A similar apical enrichment was observed for this fusion protein in the follicular epithelium (Fig. 1F). To complement this analysis, we generated a GFP probe (WASp-CRIB::GFP) that binds to active, GTP loaded Cdc42 (Fig. 1G). In photoreceptors, WASp-CRIB::GFP showed a cytosolic staining and an enrichment at the apical membrane and ZA (Fig. 1H). A mutant version of the probe that cannot bind GTP-Cdc42 (WASp-CRIB::GFP^MUT^) (Fig. 1G) showed a cytosolic staining similar to that detected with WASp-CRIB::GFP, but lacked the membrane staining (Fig. 1I). This control indicates that the cytoplasmic signal detected by WASp-CRIB::GFP is non-specific and that the apical and ZA signal is specific. The signal-to-noise ratio did not allow for detection of GTP-Cdc42 in the follicular epithelium.

**Fig. 2.**
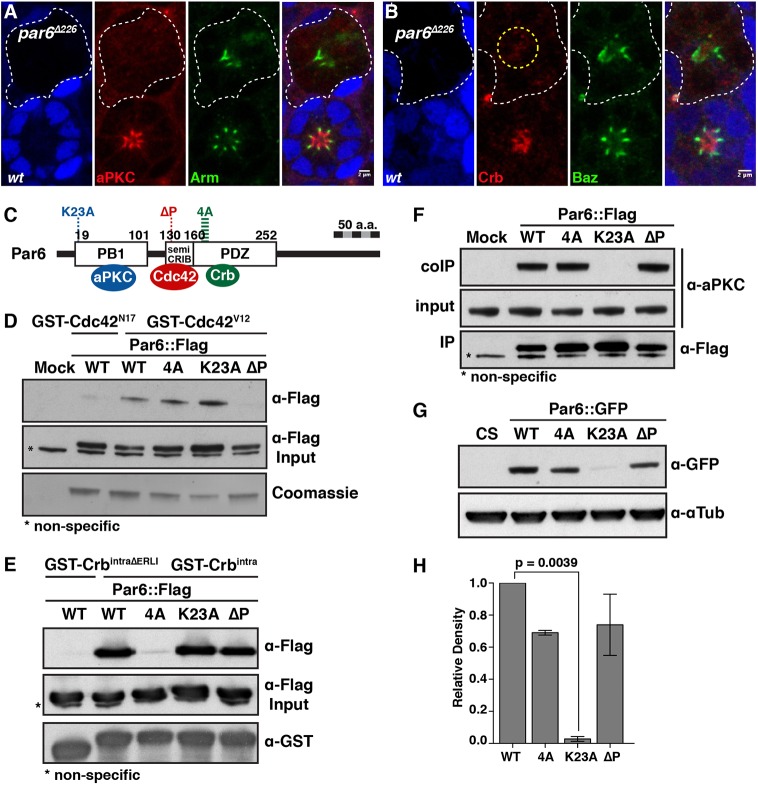
**Uncoupling Par6 from Cdc42, aPKC and Crb.** (A,B) *par6^Δ226^* mutant photoreceptors labelled by loss of GFP (blue) and stained for aPKC (A, red), Arm (A, green), Crb (B, red) and Baz (B, green). A dashed yellow circle indicates punctate structures, stained for Crb, in the presumptive apical region of the *par6* mutant cells. Scale bars: 2 µm. (C) Schematic representation of Par6, indicating the protein domains that interact with aPKC, Cdc42 and Crb, and the mutagenized sites used to uncouple these interactions. (D) GST pulldown between recombinant GST::Cdc42^V12^ and S2R^+^ cell extracts transfected with the various *par6::Flag* transgenes. Recombinant GST::Cdc42^N17^ was used as a control. (E) GST-pulldown between recombinant GST::Crb^intra^ and S2R^+^ cell extracts transfected with the various *par6::Flag* transgenes. Recombinant GST::Crb^intraΔERLI^ (Bachmann et al., 2001) was used as a control. (F) Endogenous aPKC was co-immunoprecipitated from S2R^+^ cells transfected with the various *par6::Flag* transgenes. In D and F, mock corresponds to samples transfected with empty Flag vector. (G) Western blot of protein extracts from adult heads of animals expressing the various *par6-Par6::GFP* transgenes, probed with anti-GFP and quantified in H. Wild-type Canton S (CS) fly head extracts were used as a control. Data are mean±s.e.m. from three independent experiments.

Next, we assessed whether apical localization of Cdc42 depends on Baz, Par6-aPKC and Crb. We found that mCherry::Cdc42 accumulated in cells lacking Par6 (Fig. 1J), which also lack aPKC and Crb (Fig. 2A,B). Consistent, with these results, mCherry::Cdc42 was also detected at the apical membrane of *crb* mutant cells (Fig. 1K). In addition, in *baz* mutant cells, mCherry::Cdc42 was detected in membrane domains that are apical to Arm (Fig. 1L). These results show that apical accumulation of Cdc42 does not depend on Baz, Par6-aPKC or Crb, and suggest that Cdc42 localization defines apical identity in the photoreceptor.

### Par6 is required for apical recruitment of aPKC and Crb

To further investigate the relationship between Cdc42 and the localization of the Par complex and Crb, we studied *par6* function in more detail. *par6* is required for cell viability in the retina, but as for *cdc42*, small clones of mutant cells can be recovered by raising the animals at low temperature (18°) (Walther and Pichaud, 2010). To ensure that these mutant cells were not undergoing apoptosis at the time when we examined them, we stained for Caspase 1 (Fig. S2A,B). In the absence of *par6*, aPKC and Crb were not detected at the plasma membrane of the photoreceptor (Fig. 2A,B). Instead Crb staining showed punctate structures in the apical region of the cytosol (Fig. 2B). Baz and Arm domains were still present toward the apical pole of the cells (Fig. 2A,B). Therefore, Par6 is required for aPKC and Crb recruitment at the apical membrane, but apical identity remains in its absence.

### Dissecting Par6 function during morphogenesis

Next, to probe the interface between Par6, Cdc42, aPKC and Crb, we used directed mutagenesis to individually uncouple binding between Par6 and these proteins (Fig. 2C and Fig. S1A). To disrupt Par6 binding to Cdc42, we used Par6^Δ139P^ (Par6^ΔP^), a mutated protein that cannot bind to Cdc42 (Hutterer et al., 2004). Wild-type Par6 binds to a constitutively active version of Cdc42 (Cdc42V12), but not to inactive Cdc42 (Cdc42N17) (Fig. 2D). In these pull-down experiments, interrupting Par6 binding to active Cdc42 did not interfere with the ability of this protein to bind to Crb or aPKC (Fig. 2D-F). To disrupt Par6 binding to Crb, we generated Par6^KPLG170-173AAAA^ (Par6^4A^) (Joberty et al., 2000; Li et al., 2010; Peterson et al., 2004; Whitney et al., 2016), a protein that retained its ability to bind to Cdc42 and aPKC, but cannot bind to the C-terminal ERLI PDZ-binding motif of Crb (Fig. 2D-F). Finally, to uncouple Par6 from aPKC, we generated Par6^K23A^ (Noda et al., 2003), in which Par6 binding to aPKC is abolished without affecting its binding to Cdc42 or Crb (Fig. 2D-F).

All Par6 transgenes were GFP tagged and placed under the control of a 1 kb minimal *par6* promoter to generate transgenic animals. Western blotting from fly head extracts showed that all fusion proteins, except for Par6^K23A^, could be detected and were expressed at similar levels (Fig. 2G,H). Because Par6^K23A^ could be stably expressed in S2R^+^ cells (Fig. 2D-F), our results suggest that aPKC binding to Par6 is required to stabilize Par6 *in vivo*. Expressing *par6-Par6::GFP* in *par6* mutant animals supported animal viability. This is in contrast to *par6-Par6*^Δ*P*^*::GFP* and *par6-Par6^K23A^::GFP*, which failed to support viability when expressed in *par6* mutant animals. When *par6-Par6^4A^::GFP* was expressed, a few adult males homozygous mutant for *par6* could be recovered. However, these males were observed at less than the expected Mendelian frequency and were infertile.

### aPKC binding to Par6 is required for the apical localization of Par6

**Fig. 3.**
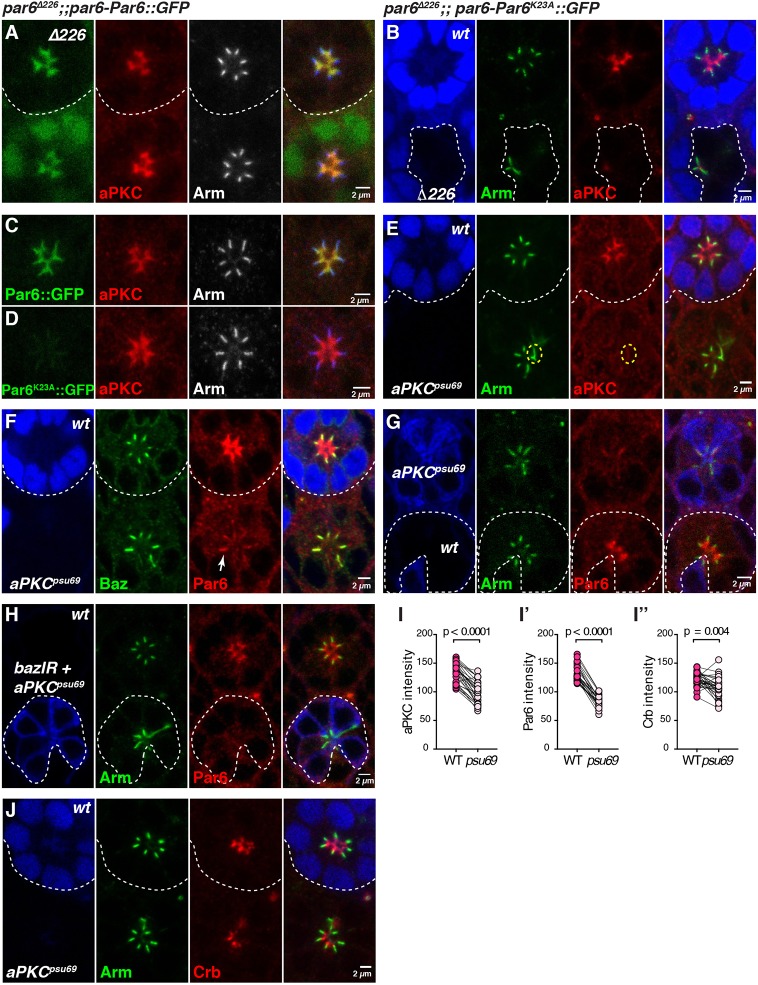
**aPKC binding is essential for the apical accumulation of Par6.** (A,B) *par6*^Δ*226*^ mutant cells expressing *par6-Par6::GFP* (A) or *par6-Par6^K23A^::GFP* (B). In A, the mutant cells are labelled by loss of nuclear GFP signal (green) and stained for aPKC (red) and Arm (grey, blue in the merged channel). In B, the mutant cells expressing *par6-Par6^K23A^::GFP* are identified by the loss of GFP (blue) and are stained for Arm (green) and aPKC (red). (C) *par6-Par6::GFP* (green) expressed in an otherwise wild-type ommatidium and stained for aPKC (red) and Arm (grey; blue in the merged channel). (D) *par6-Par6^K23A^::GFP* (green) expressed in an otherwise wild-type ommatidium and stained for aPKC (red) and Arm (grey; blue in the merged channel). (E,F) *aPKC^psu69^* mutant photoreceptors lacking nuclear GFP (blue) and stained for Arm (E, green), aPKC (E, red), Baz (F, green) and Par6 (F, red). In E, a yellow dashed line encircles an apical region where Arm and aPKC overlap. In F, Par6 localizes predominantly at the ZA (white arrow). (G) *aPKC^psu69^* mutant photoreceptors positively marked by cytosolic GFP (blue), and stained for Arm (green) and Par6 (red). (H) *aPKC^psu69^* cells (blue) co-expressing *bazIR* and stained for Arm (green) and Par6 (red). (I-I″) Quantification of aPKC, Par6 and Crb intensity at the membrane of *aPKC^psu69^* mutant photoreceptors, compared with neighbouring wild-type photoreceptors. (J) *aPKC^psu69^* mutant photoreceptors lacking nuclear GFP (blue) and stained for Arm (green) and Crb (red). Scale bars: 2 µm.

To test the suggestion that aPKC binding to Par6 is required to stabilize Par6 *in vivo*, we examined the expression of the *par6-Par6^K23A^::GFP* transgene in the photoreceptor. Reintroducing Par6::GFP in *par6*^Δ*226*^ or *par6^29VV^* mutant cells fully rescued the loss of aPKC and Crb at the apical membrane, and ZA positioning along the apical-basal axis (Fig. 3A and Fig. S3A, and not shown). In contrast, *par6-Par6^K23A^::GFP* failed to rescue the *par6* mutant phenotype (Fig. 3B), and Par6^K23A^ was detected at very low levels at the ZA when expressed in otherwise wild-type cells (Fig. 3C,D). To complement this analysis, we made use of the *aPKC^psu69^* allele, which encodes a version of aPKC that does not bind to Par6 (Kim et al., 2009). In *aPKC^psu69^* mutant cells, only very low levels of aPKC were detected at the ZA associated with Arm (Fig. 3E, quantified in 3I). Similarly, Par6 did not accumulate at the apical membrane and instead was also found at low levels at the ZA (Fig. 3F,G, quantified in 3I′). Furthermore, in *aPKC^psu69^* mutant cells, Par6 localization at the ZA was dependent on *baz* (Fig. 3H). However, Crb levels were only marginally lower than in wild-type cells (Fig. 3J and quantified in 3I″). Altogether, these results show that, in the photoreceptor, aPKC binding to Par6 is required to promote the apical localization and accumulation of Par6-aPKC. However, it is not required for the recruitment of Crb, Par6 and aPKC at the plasma membrane, and our results suggest Par6 and aPKC can be localized to the photoreceptor ZA through Baz.

### Cdc42 regulates the apical localization of Par6

**Fig. 4.**
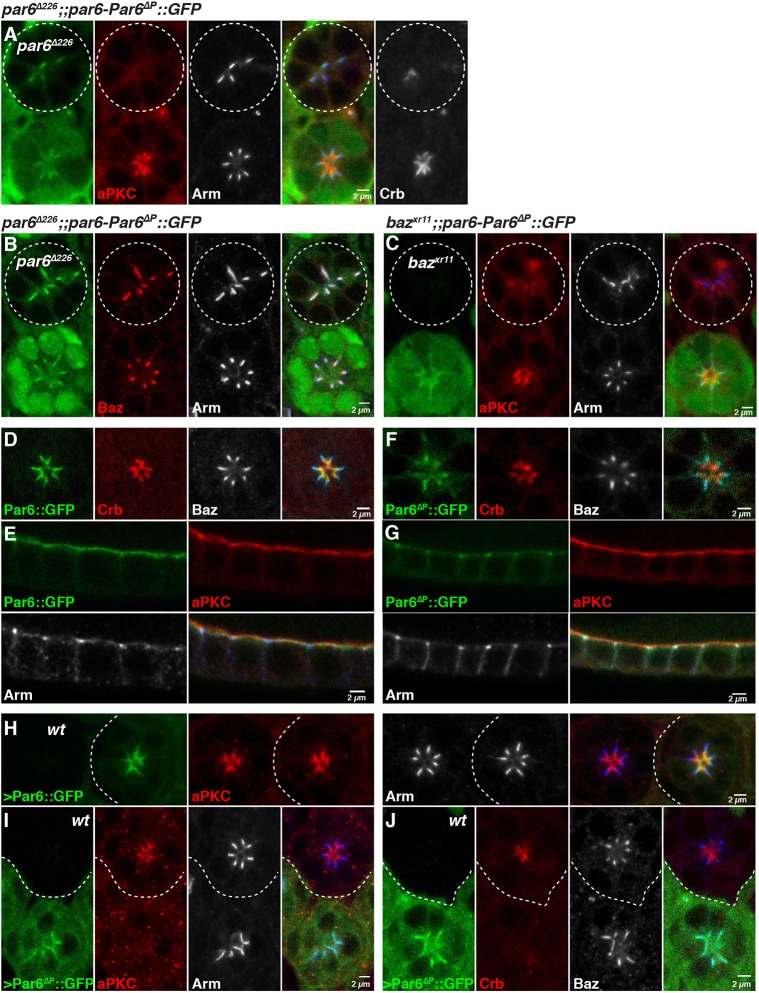
**Cdc42 regulates the apical localization of Par6.** (A,B) *par6*^Δ*226*^ mutant photoreceptors and expressing *par6-Par6*^Δ*P*^*::GFP* (green, circled). *par6*^Δ*226*^ mutant cells are labelled by loss of nuclear GFP signal (green) and stained for aPKC (A, red), Baz (B, red) and Arm (grey). In A, an additional panel is included showing Crb staining in grey that is not part of the merged panel. (C) *baz^XR11^* mutant photoreceptors expressing *par6-Par6*^Δ*P*^*::GFP* (green, circled). *baz^XR11^* mutant cells are labelled by loss of nuclear GFP signal and stained for aPKC (red) and Arm (grey). (D,E) *par6-Par6::GFP* (green) expressed in otherwise wild-type (D) photoreceptors (Crb, red and Baz, grey) and (E) follicular epithelial cells (aPKC, red; Arm, grey). (F,G) *par6-Par6*^Δ*P*^*::GFP* expressed in otherwise wild-type (F) photoreceptors (Crb, red; Baz, grey) and (G) follicular epithelial cells (aPKC, red; Arm, grey). Note that in F the *par6-Par6*^Δ*P*^*::GFP* transgene was imaged using increased laser power relative to that used for the wild-type transgene. (H-J) Overexpression of (H) wild-type Par6::GFP (green) and (I,J) Par6^ΔP^::GFP (green) in mosaic retinas. In all cases, the non-expressing cells are labelled ‘wt’. Cells are stained for (H,I) aPKC (red) and Arm (grey); and (J) Crb (red) and Baz (grey). In all panels except for A, the grey channel is shown in blue in the merge. Scale bars: 2 µm.

To better understand how Cdc42 binding to Par6 regulates epithelial morphogenesis, we made use of the *par6-Par6*^Δ*P*^*::GFP* transgene. We found that while this transgene failed to rescue the *par6* loss of function in the photoreceptor, the Par6^ΔP^::GFP mutant protein was detected toward the apical pole of the cell, where it colocalized with Baz and Arm (Fig. 4A,B and Fig. S3B,C). Very low levels of aPKC were detected in domains containing both Arm and Par6^ΔP^::GFP (Fig. 4A). However, we noted instances where aPKC staining could be detected immediately apical to these domains (Fig. S3B). In addition, we found that the recruitment of Par6^ΔP^::GFP at the membrane required *baz* (Fig. 4C)*.* This finding is consistent with Par6 binding to Baz (Renschler et al., 2018).

To further assess the effect of uncoupling Par6 from Cdc42, we expressed *par6-Par6^ΔP^::GFP* in otherwise wild-type cells. In the photoreceptor and follicular epithelium, Par6::GFP localized as endogenous Par6 (Fig. S1C,G, Fig. 4D,E and Fig. S3D,E,H). In contrast, Par6^ΔP^::GFP was found at low levels at the developing ZA (Fig. 4F,G and Fig. S3F,G,I, quantified in S3J). We noted that in both the photoreceptor and follicular epithelium, Par6^ΔP^::GFP failed to localize at the apical membrane, even though Crb was present (Fig. 4F and Fig. S3G,I). These results indicate that Cdc42 binding to Par6 is required for localization and accumulation of Par6 apical to the ZA.

To further probe the relationship between Par6 and aPKC and Crb, we overexpressed Par6^ΔP^::GFP using the UASp-Par6^ΔP^::GFP transgene. Overexpression of wild-type Par6::GFP had no discernible effect on the localization of apical and ZA proteins (Fig. 4H). In contrast, overexpression of Par6^ΔP^::GFP led to a strong decrease in the apical levels of aPKC and Crb when compared with the neighbouring wild-type cells. In addition, Par6^ΔP^::GFP was colocalized with Arm and Baz, and detected in the cytosol (Fig. 4I,J). These effects on aPKC and Crb accumulation likely reflect Par6^ΔP^::GFP outcompeting endogenous Par6 at the plasma membrane. They confirm that Cdc42 binding to Par6 regulates the apical accumulation of aPKC, and support the hypothesis that, together, Cdc42, Par6 and aPKC promote the apical accumulation of Crb.

### Crb is required to deplete Par6-aPKC from the developing ZA

**Fig. 5.**
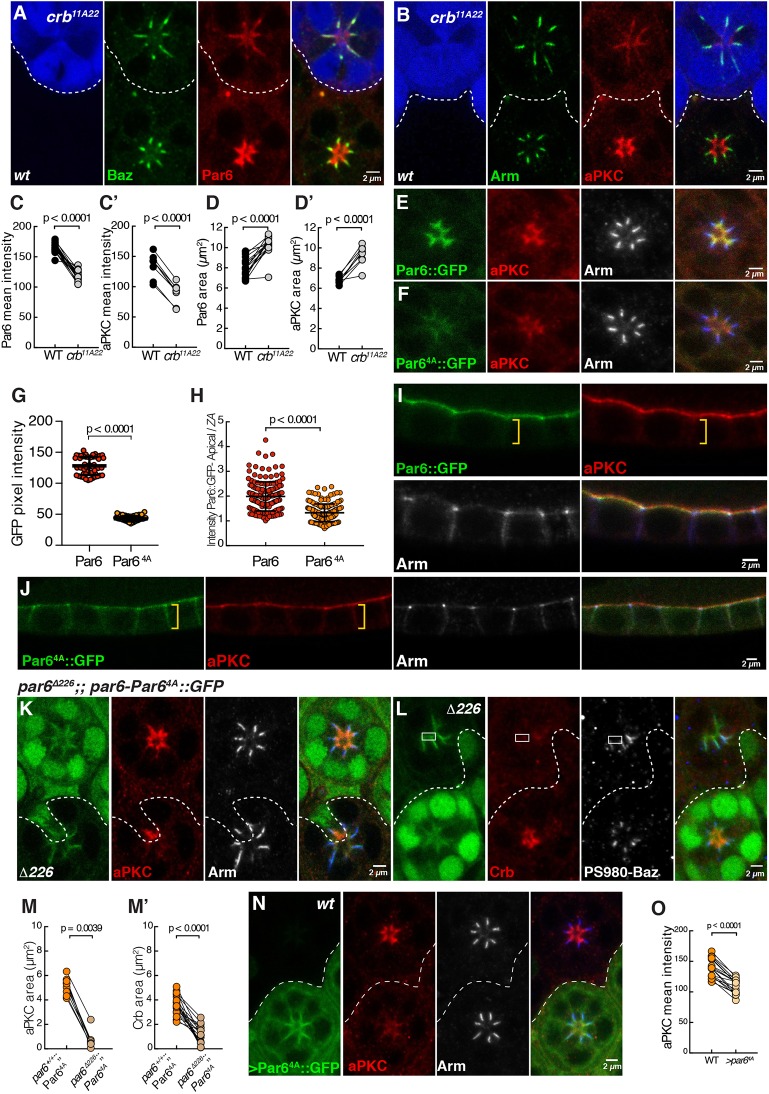
**Crb is required to deplete Par6-aPKC from the developing ZA.** (A,B) *crb^11A22^* mutant photoreceptors positively labelled by GFP (blue) and stained for Baz (A, green), Par6 (A, red), Arm (B, green) and aPKC (B, red). (C,C′) Par6 and aPKC mean intensity and (D-D′) Par6 and aPKC area quantifications in *crb^11A22^* mutant photoreceptors compared with wild type. Twenty ommatidia pairs from seven retinas and nine ommatidia pairs from three retinas were measured for Par6 and aPKC, respectively. (E) *par6-Par6::GFP* (green) expressed in otherwise wild-type photoreceptors labelled for aPKC (red) and Arm (grey). (F) *par6-Par6^4A^::GFP* (green) expressed in otherwise wild-type photoreceptors labelled for aPKC (red) and Arm (grey). Both E and F were imaged at the same microscope settings. (G) Quantification of *par6-Par6::GFP* and *par6-Par6^4A^::GFP* mean pixel intensity in photoreceptors. (H) Ratio of the GFP intensity measured at the apical membrane to that measured in the ZA for Par6::GFP and Par6^4A^::GFP. At least 160 ratios were calculated from at least three retinas per genotype. (I) *par6-Par6::GFP* (green) and (J) *par6-Par6^4A^::GFP* (green) expressed in otherwise wild-type follicular epithelial cells stained for aPKC (red) and Arm (grey). A yellow bracket indicates the lateral membrane. (K,L) *par6* mutant photoreceptors lacking nuclear GFP signal (green), expressing *par6-Par6^4A^::GFP* (green) and stained for aPKC (K, red), Arm (K, grey), Crb (L, red) and Phospho-S980-Baz (L, grey). In L, a rectangle indicates *par6-Par6^4A^::GFP* signal detected immediately apical to P-S980Baz. (M,M′) Quantification of aPKC and Crb areas upon overexpression of Par6^4A^::GFP. Nine ommatidia pairs from four retinas and 19 ommatidia pairs from six retinas were analysed for aPKC and Crb, respectively. (N) Photoreceptors overexpressing Par6^4A^::GFP (green), stained for aPKC (red) and Arm (grey). (O) Quantification of aPKC mean intensity at the apical membrane of neighbouring wild-type and Par6^4A^::GFP-overexpressing cells. Where appropriate, the grey channel is shown in blue in the merged panel. Scale bars: 2 µm.

To further probe the relationship between Crb and Par6, we generated *crb^11A22^* mutant cells. In the absence of *crb*, a reproducible apical fraction of Par6 and aPKC is separated from the ZA containing Baz and Arm (Fig. 5A,B). Quantification of the Par6 and aPKC signals along the apical-basal axis showed that the amount of these proteins at the apical membrane is lower than in wild-type cells (Fig. 5C,C′). Furthermore, these two proteins spread towards the basal pole of the cells (Fig. 5A,B,D,D′).

To complement our analysis of the *crb* mutant phenotype, we made use of the *par6-Par6^4A^::GFP* transgene. When expressed in otherwise wild-type cells, the apical levels of Par6^4A^::GFP were lower than those measured for Par6::GFP (Fig. 5E,F, quantified in 5G) and the GFP signal invaded the ZA (Fig. 5H). These results support our model that Par6 binding to Crb is required for the accumulation of Par6 at the apical membrane. In addition, examination of this transgene in the follicular epithelium revealed that it spread to the lateral membrane, suggesting that binding of Par6 to Crb can serve as an apical retention mechanism (Fig. 5I,J). Next, we assessed the ability of Par6^4A^::GFP to rescue the failure of aPKC and Crb apical recruitment observed in *par6*^Δ*226*^ mutant photoreceptors. We found that Par6^4A^::GFP cannot support the apical accumulation of aPKC and Crb (Fig. 5K,L, quantified in 5M,M′). Par6^4A^::GFP was detected at the apical pole of the cells, with low levels at the apical membrane and higher levels colocalized with adherens junction material, including P-S980-Baz (Fig. 5K,L).

Finally, overexpressing Par6^4A^::GFP under the control of a UAS promoter revealed apical accumulation of aPKC was reduced, which is compatible with Par6^4A^::GFP binding to aPKC but not being able to accumulate at the membrane (Fig. 5N, quantified in 5O). UAS-Par6^4A^::GFP was also detected in the cytosol (Fig. 5N). Altogether, our results show that an essential contribution of Par6 binding to Crb is to promote the apical retention and accumulation of Par6-aPKC and that of Crb at the developing apical membrane.

### Par6 and Crb can accumulate at the apical membrane when Sdt levels are reduced

**Fig. 6.**
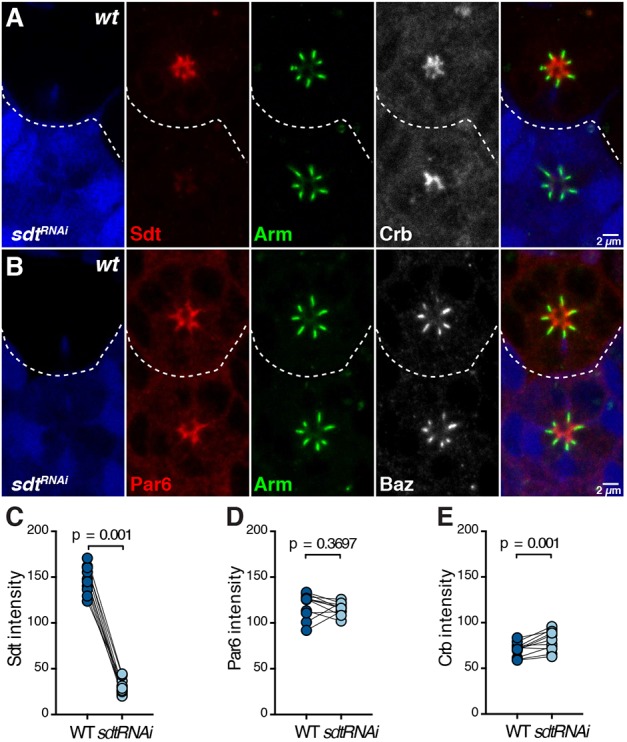
**Par6 and Crb can accumulate at the apical membrane when Sdt levels are reduced.** (A,B) *SdtIR* cells (GFP, blue) stained for Sdt (A, red), Arm (green), Crb (A, grey), Par6 (B, red) and Baz (B, grey). In A and B, the grey panels shown are not included in the merged panels. Scale bars: 2 µm. (C-E) Quantification of Sdt (C), Par6 (D) or Crb (E) intensity at the apical membrane in *SdtIR* and neighbouring wild-type cells.

Par6 interaction with Crb could be direct, or indirect through Sdt. We have previously shown that Sdt is largely dispensable for the apical localization of Par6 and aPKC early during photoreceptor morphogenesis (Walther and Pichaud, 2010). To complement this previous analysis, we made use of RNAi in mosaic retinas and examined the expression of Crb and Par6. Using this approach, quantification revealed that *sdt* levels were decreased by up to 90% when compared to neighbouring wild-type cells (Fig. 6A,C). However, in these cells, we did not detect any significant change in Par6 accumulation at the apical membrane (Fig. 6B,D). A modest increase in Crb apical accumulation was detected within cells expressing Sdt RNAi when compared with neighbouring wild-type cells (Fig. 6A,E). These results, together with those of Walther and Pichaud (2010), show that, *in vivo*, Par6 and Crb can accumulate when Sdt levels are strongly reduced. They are compatible with apical localization of Par6 resulting from direct binding to Crb.

### The Crb-binding domain in Par6 is important for Par6 stability at the apical membrane

**Fig. 7.**
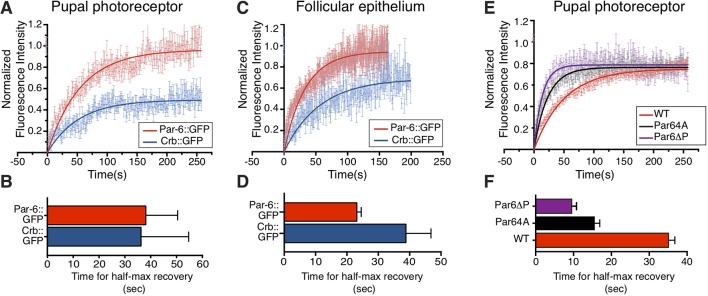
**Crb promotes apical retention of Par6.** (A-D) FRAP of Crb::GFP (blue) and par6-Par6::GFP (red) in the developing photoreceptor (A,B) and the follicular epithelia (C,D). For Par6, the *par6-par6::GFP* transgene is expressed in otherwise wild-type photoreceptors. In the case of the follicular epithelium, *par6-par6::GFP* was expressed in *par6* mutant cells. Fluorescence recovery curves were calculated using a single exponential fit of the FRAP data. The half-time recoveries of Crb::GFP (blue) and Par6::GFP in the photoreceptor and follicular epithelium are shown in B and D, respectively. (E) FRAP of Par6::GFP, Par6^4A^::GFP and Par6^ΔP^::GFP overexpressed using the Gal4-UAS system. The graph shows mean normalized fluorescence intensity for Par6::GFP (red, *n*=14 from three retinas), Par6^4A^::GFP (grey, *n*=16 from three retinas) and Par6^ΔP^::GFP (purple, *n*=13 from two retinas). Data are mean±s.e.m. Fluorescence recovery curves were calculated using a single exponential fit. (F) Half-time recovery of Par6::GFP (red), Par6^4A^::GFP (black) and Par6^ΔP^::GFP (purple).

One possible explanation for our observation that the Crb-Par6 interface is required for Par6 apical accumulation is that Par6 binding to Crb stabilizes Par6 at the apical membrane. To test this, we used fluorescence recovery after photobleaching (FRAP) and assessed the recovery rate of Par6 and Par6^4A^::GFP in photoreceptors and the follicular epithelium. FRAP experiments of *par6-Par6::GFP* expressed in otherwise wild-type cells over ∼4 min show that up to 95% of Par6 is mobile, with a t_1/2_ of ∼40 s in the photoreceptor (Fig. 7A,B and Fig. S4A). We found a similarly high mobile fraction for Par6::GFP in the follicular epithelium, but the estimated t_1/2_ of ∼25 s is shorter than that measured in the retina (Fig. 7C,D and Fig. S4B). These results show that, overall, most of Par6 is mobile over a period of 4 min in both epithelia.

Expression of Par6^4A^ under the minimal *par6* promoter leads to a weak apical accumulation (Fig. 5F), which necessitated the use of the UAS-Gal4 system for further FRAP analysis. In the photoreceptor, the mobile fraction (Fig. 7E and Fig. S4C) and the t_1/2_ of recovery (Fig. 7F) for UAS-Par6::GFP were comparable with that of *par6-*Par6::GFP (Fig. 7A,B). Similar mobile fractions (∼80%) were estimated for wild-type UAS-Par6 and UAS-Par6^4A^ (Fig. 7E). However, Par6^4A^ recovered twice as fast in comparison with Par6 wild type, with a t_1/2_ of ∼35 s for UAS-Par6::GFP compared with a t_1/2_ of ∼15 s for UAS-Par6^4A^::GFP (Fig. 7F). These results suggest that Crb can either limit the lateral diffusion of Par6 or regulate its on/off rate at the apical membrane. In parallel, we estimated the mobile fraction and t_1/2_ for the fraction of Par6^ΔP^::GFP associated with adherens junction material when overexpressed in the photoreceptors. We found that the mobile fraction of this protein is similar to that of Par6 and Par6^4A^::GFP (Fig. 7E). We estimate the t_1/2_ of UAS-Par6^ΔP^::GFP to be ∼10 s (Fig. 7F). This indicates that Par6 binding to Cdc42 is required to stabilize Par6.

To complement this analysis, we also performed FRAP on endogenously tagged Crb (Crb::GFP). In the photoreceptor, we found that over a period of ∼4 min, approximately 40% of Crb is mobile (Fig. 7A). The mobile fraction recovers with a t_1/2_ of ∼40 s, a value that is similar to that estimated for the Par6 mobile fraction over the same time span (Fig. 7B). In the follicular epithelium, we found that up to 60% of Crb::GFP is mobile, with a t_1/2_ of ∼40 s (Fig. 7C-D). Overall, our results indicate that a greater fraction of Crb is stable at the apical membrane of these two epithelial cell types when compared to Par6.

## DISCUSSION

**Fig. 8.**
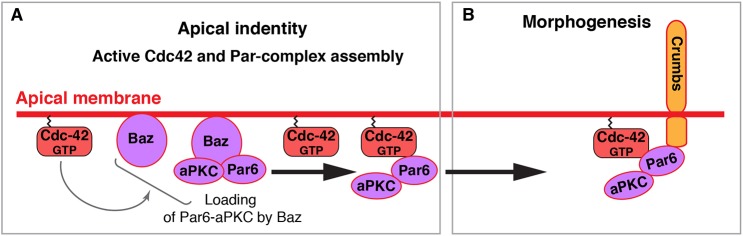
**Apical identity and morphogenesis in the photoreceptor.** (A) Defining the apical pole of the cell. Active Cdc42 is detected at the apical membrane (this work) and is required for Baz-Par6-aPKC localization at the membrane (this work and Walther and Pichaud, 2010). This requirement is indicated with the grey arrow from Cdc42 towards Baz and Baz-Par6-aPKC. The nature of the requirement for Cdc42 in promoting Baz membrane localization is not known. In this model, Baz loads Par6-aPKC (Par complex) at the membrane. Par6-aPKC exchanges from Baz to Cdc42 between the Par complex and the ternary Cdc42-Par6-aPKC complex. (B) Epithelial morphogenesis. Cdc42 binding to Par6 promotes the retention of Par6-aPKC by Crb (this work). In this model, apical delivery of Crb coincides with active Cdc42 at the apical pole of the cell. Binding of Par6 to Crb promotes the stabilization of Crb at the membrane and apical retention of Par6-aPKC. Apical accumulation of Crb is required for photoreceptor morphogenesis (Izaddoost et al., 2002; Pellikka et al., 2002).

How Cdc42, the Par complex (Baz-Par6-aPKC), Crb/CRB3 and Sdt/PALS1 work together to regulate epithelial morphogenesis is not well understood. Our work in the developing photoreceptor indicates that Baz is required to load Par6 and aPKC at the apical pole of the cell (Walther et al., 2016; Walther and Pichaud, 2010). Here, we present evidence that apical accumulation of Cdc42 occurs independently of these Par-complex components and Crb. Furthermore, our results show that Par6-aPKC can localize apical to the ZA in the absence of Crb, presumably through binding to Cdc42 or Baz. Our work also indicates that at the apical pole the fraction of Par6-aPKC that is associated with Cdc42 is captured by Crb. We show evidence that this capture mechanism depends on Par6 binding to Crb, and on the association of Par6 with aPKC. In turn, Par6 binding to Crb promotes the accumulation of this transmembrane factor. Thus, Par6-aPKC and Crb mutually reinforce each other's apical localization and accumulation to support apical membrane and ZA morphogenesis. Altogether, our work supports a model whereby coinciding apical accumulation of Cdc42, loading of Par6-aPKC through Baz and apical delivery of Crb define the apical pole of the cell and promote apical membrane and ZA morphogenesis. In this model, the ability of Par6 to bind to Cdc42 and Crb links Cdc42-dependent apical identity to Par6-aPKC recruitment and apical accumulation of Crb. Our model is summarized in Fig. 8.

### Cdc42 defines apical identity and promotes morphogenesis through Par6-aPKC

Cdc42 accumulates at the apical pole of the photoreceptor in the absence of Baz, Par6, aPKC and Crb. Therefore, we hypothesize that apical recruitment of Cdc42 is regulated by a route that does not depend on these polarity factors. We envisage a model whereby one or several Cdc42 GEFs activate and thus promote apical accumulation of Cdc42. In this model, Cdc42 defines the apical pole of the cell and enables morphogenesis through Par6-aPKC and Crb. Considering the interface between Cdc42 and Par6, our results indicate that Par6 binding to Cdc42 is required for Par6 to be localized at the apical membrane. In the photoreceptor, Par6^ΔP^::GFP colocalizes with adherens junction material*.* Furthermore, its expression in otherwise wild-type cells leads to its localization at the ZA, and this localization depends on *baz*. These findings differ from the situation reported in the embryonic ectoderm, where a similar (untagged) version of Par6 failed to be recruited at the membrane altogether (Hutterer et al., 2004). Furthermore, our finding that low levels of Par6 and aPKC localize with Baz at the ZA when binding between these two proteins has been interrupted genetically using the *aPKC^psu69^* allele, raises the possibility that Baz can recruit Par6 and aPKC independently. A similar situation is seen in the cellularizing embryo, where Baz has been shown to support aPKC localization independently of Par6 (Harris and Peifer, 2005).

### Par6-aPKC distributes between the Par- and Cdc42-Par6-aPKC complexes

While Baz promotes Par6-aPKC localization at the apical membrane of the photoreceptor, very low levels of these proteins can still be detected in a fraction of *baz* mutant cells (Walther et al., 2016). This indicates that Par6-aPKC can be recruited independently of Baz, presumably through binding to Cdc42 or Crb. In addition, in the absence of Crb, we can still detect Par6-aPKC at the membrane, apical to Baz domains. Furthermore, our rescue experiments expressing the version of Par6 that cannot bind to Crb show that Par6-aPKC can localize apical to ZA-like domains. Altogether, these results lead us to hypothesize that the apical fraction of Par6-aPKC we detect in *crb* mutant cells is bound to either Baz or Cdc42. In wild-type cells, the fraction that is associated with Cdc42 is captured by Crb, and our results show that binding of Cdc42 to Par6 is required for separating Par6 from Baz. Therefore, we propose that Par6-aPKC can distribute between the Par complex and a ternary Cdc42-Par6-aPKC complex that promotes apical capture of Par6 by Crb. This situation bears a striking resemblance to that recently reported in the *C. elegans* zygote, where PAR-3, PAR-6 and PKC-3 define antero-posterior polarity (Rodriguez et al., 2017; Wang et al., 2017). In this cell, the Par complex has been shown to exist next to a ternary CDC-42-PAR-6-PKC-3 complex (Rodriguez et al., 2017; Wang et al., 2017). Further work will be required to elucidate exactly how these two complexes relate to each other in epithelial cells.

### Cdc42 enables the apical retention of Par6-aPKC by Crb

In both the photoreceptor and follicular epithelium, we find that although binding of Par6 to Crb is not required to localize Par6-aPKC at the apical membrane, it is required to promote the apical accumulation of these factors. We were able to recover a few viable males of the *par6-Par6^4A^::GFP* genotype, indicating that binding of Par6 to Crb is not absolutely required for animal viability. Presumably, maternal contribution provides enough Par6 to overcome embryonic development and, in rare cases, the Par6^4A^::GFP protein is sufficient to complete development to adulthood. Nevertheless, when considering the interface between Par6 and Crb, previous studies raise the possibility that Par6-aPKC can be recruited by Crb either directly or indirectly through Sdt (Lemmers et al., 2004). The Par6^4A^ transgene we have generated here disrupts the PDZ domain, which supports binding of Par6 to both Crb and Sdt. Therefore, this transgene alone does not allow us to distinguish between these two possibilities. However, our past work (Walther and Pichaud, 2010), that of others (Hong et al., 2003) and present analyses of *sdt*-deficient photoreceptors indicates that Par6, aPKC and Crb can be detected when Sdt expression is abolished or strongly reduced. These results argue that Crb can support Par6-aPKC apical accumulation in the absence of Sdt. They support a model whereby direct binding of Par6 onto Crb mediates the apical retention and accumulation of Par6-aPKC. This model is consistent with the finding that Cdc42 enhances binding of Par6 to the Crb PDZ-binding domain, but does not enhance binding to the ECR1 motif of Sdt (Whitney et al., 2016). Further, our finding that Crb levels are marginally increased when Sdt expression is decreased using RNAi is consistent with recent work showing that Sdt isoforms can promote the endocytosis of Crb *in vivo* (Perez-Mockus et al., 2017). Interestingly, previous work has shown Crb is absent from the subapical membrane (stalk membrane) of mature photoreceptors in *sdt* mutant cells (Berger et al., 2007). Together with our work, this indicates that when considering Crb apical localization the requirement for Sdt differs depending on the developmental stage.

Par6 binding to Crb promotes the accumulation of Par6-aPKC and Crb at the apical pole of the cell. Consistent with this model, our FRAP experiments show that the t_1/2_ recovery of the Par6^4A^ mobile fraction is half of that of wild-type Par6. Assuming that Par6 mobile fraction is both a function of lateral diffusion and on/off rate, these results suggest that Crb binding limits one or both of these parameters for Par6. We also find that over 4 min, up to 95% of Par6 is mobile at the apical membrane. Over the same time span, ∼40% of Crb is mobile. Therefore, a larger fraction of Crb is stable at the membrane when compared with Par6. This is likely to be linked to the fact that Crb is a transmembrane protein that requires endocytosis and recycling for its turnover. Further work will be needed to better understand what these fractions are and how they are regulated.

## MATERIALS AND METHODS

### Fly strains

The following fly strains were used:

*par6^Δ226^, FRT9.2* is an amorphic allele (Petronczki and Knoblich, 2001);

*par6^29VV^, FRT19A* is an amorphic allele (Jones and Metzstein, 2011);

*w, baz^xi106^, FRT9.2* (Nusslein-Volhard et al., 1987);

*w, baz^XR11^, FRT19A* (Shahab et al., 2015);

*w ; FRTG13, aPKC^psu69^* (Kim et al., 2009);

*w ;; FRT82B, crb^11A22^* (Tepass et al., 1990);

*w ;; Crb::GFP* (Huang et al., 2009);

*w ; (sqh-ChFP-Cdc42)^23^* and *w ;; (sqh-ChFP-Cdc42)^33^* (Abreu-Blanco et al., 2014);

*w, cdc42^3^, FRT9.2* (Genova et al., 2000) (*cdc42^3^* is a G to A mutation resulting in an amino acid substitution of G114 for Asp);

*w, cdc42^4^, FRT19A* (Fehon et al., 1997) (*cdc42^4^* is a G to A mutation that results in the mutation of a splice acceptor site);

*w ;; cdc42^IR^* [Vienna *Drosophila* Resource Center (VDRC) 100794];

*w ; baz^IR^* [Bloomington *Drosophila* Stock Center (BDSC) 35002];

*w ;; sdt^IR^* [Bloomington *Drosophila* Stock Center (BDSC) 33909]; and

*w ;* *GMR-Gal4* (Freeman, 1996).

The following fly strains were generated in this study: *w* ;; *par6-Par6::GFP*, *w* *;; par6-Par6^K23A^::GFP*, *w* ;; *par6-Par6^ΔP^::GFP*, *w* ;; *par6-Par6^4A^::GFP*, *w ;; UASp-Par6::GFP*, *w ;; UASp-Par6^4A^::GFP*, w *;;* UASp-Par6^ΔP^::GFP. UASp-WASp-CRIB::GFP and UASp-WASp-CRIB::GFP^MUT^. To generate *par6*-*Par6::GFP* rescue strains, the appropriate DNA constructs were injected into the parent strain *y^1^, w^67c23^ ;; P{Cary} attP2* [Bloomington *Drosophila* Stock Centre (BDSC) 8622] for PhiC31-mediated recombination (Groth et al., 2004) by BestGene. UASp-Par6::GFP (wild type, ΔP and 4A), UASp-WASp-CRIB::GFP and UASp-WASp-CRIB::GFP^MUT^ strains were generated by injecting the appropriate DNA constructs for standard P-element transformation (BestGene) (Rubin and Spradling, 1982).

### Molecular biology

*par6* cDNA (Clone LD29223) was obtained from the *Drosophila* Genomics Resource Center (Indiana, USA) and cloned into the *pENTR™/D-TOPO* vector (Invitrogen) to generate *pENTR-Par6* cDNA. The *par6* genomic construct, *pENTR-H427* was a kind gift from Jurgen Knoblich (Institute of Molecular Biotechnology of the Austrian Academy of Sciences). *pENTR-H427* contains a 1 kb minimal *par6* promoter (∼1 kb region upstream of the ATG start codon), the *par6*-coding region with a C-terminal GFP fusion and a minimal *par6* 3′UTR. Following sequence verification of both *par6* pENTR starting vectors, the QuikChange Mutagenesis System (Agilent) was used to generate the Par6^K23A^, Par6^ΔP^ and Par6^4A^ substitutions. Par6^ΔP^ is a deletion of P139, while Par6^4A^ is the substitution of residues KPLG170-173 for alanines. The mutated *par6* pENTR vectors were sequence verified and used for cloning with the Gateway Cloning system (Invitrogen). The destination vector *pBID-G* (Addgene 35195) was used in combination with *pENTR-H427*-derived mutants to generate all *par6*-Par6::GFP rescue constructs. All other *par6* DNA constructs used in this study are derivatives of *pENTR-Par6* cDNA. The destination vector *pPWG* from the *Drosophila* Gateway Vector collection was used to generate *UASp-Par6::GFP*, *UASp-Par6*^Δ*P*^*::GFP* and *UASp-Par6^4A^::GFP* DNA constructs. For the *UASp-WASp-CRIB::GFP* and *UASp-WASp-CRIB::GFP^MUT^* constructs, the CRIB domain of *Drosophila* WASp spanning residues K226 to A313 was PCR amplified from *UASp-WASp::GFP* (a gift from Buzz Baum, Medical Research Council/University College London Laboratory for Molecular Cell Biology) and cloned into the *pENTR™/D-TOPO* vector (Invitrogen). To disrupt binding of the WASp CRIB domain to Cdc42-GTP, substitutions F240D, H242D and H245D were introduced (Tskvitaria-Fuller et al., 2006) using the QuikChange Mutagenesis System (Agilent). Following sequence verification of both constructs, the destination vector *pPWG* from the *Drosophila* Gateway Vector collection was used to generate *UASp-WASp-CRIB::GFP* and *UAS-WASp-CRIB::GFP^MUT^.*

To generate *pActin-Par6::FLAG* constructs for expression in S2R+ cells, the *pAWF* destination vector was used. *pAWF* was a gift from Nic Tapon (The Francis Crick Institute). cDNA constructs encoding *Cdc42^N17^* and *Cdc42^V12^* were generated and cloned into the *pENTR™/D-TOPO* vector (Invitrogen). The *pDEST15* vector, containing an N-terminal GST tag, was used to generate plasmids *GST::Cdc42^N17^* and *GST::Cdc42^N17^* using the Gateway Cloning System (Invitrogen). The *GST::Crb^intra^* and *GST::Crb^intraΔERLI^* constructs were provided by E. Knust (Max Plank Institute of Molecular Cell Biology and Genetics) (Kempkens et al., 2006).

### Genetics

Clonal analysis of mutant alleles in the retina was performed using either the standard FLP-FRT technique (Xu and Rubin, 1993) with appropriate *FRT, ubi-GFP* chromosomes used to generate negatively marked mutant tissue, or using MARCM (Lee and Luo, 2001) to generate positively marked mutant tissue. In both cases, eyFLP (Newsome et al., 2000) was used. MARCM was used to generate retinal tissue expressing *baz^IR^* in the *aPKC^psu69^* mutant background. Rescue experiments with *par6*-*Par6::GFP* transgenes were performed by building fly strains carrying a specified mutant chromosome and the *par6-Par6::GFP* transgene of interest, followed by FLP-FRT induction of clones. Retinal clones overexpressing UAS-Par6::GFP transgenes, UAS-WASp-CRIB::GFP or UAS-WASp-CRIB::GFP^MUT^ were generated with the coinFLP system (Bosch et al., 2015) using BDSC stock 58750.

### Immunofluorescence

Whole-mount retinas at 40% after puparium formation (APF) were prepared as previously described (Walther and Pichaud, 2006). Samples were fixed in 4% formaldehyde in PBS for 20 min and blocked in 5% goat serum in PBS with 0.3% Triton (PBST) for 20 min. All subsequent incubations were performed in PBST. Samples were incubated overnight at 4°C in primary antibodies, washed 3 times for 5 min, incubated in secondary antibodies for 4-6 h and washed overnight at 4°C. Ovaries were dissected in PBS, fixed in 4% formaldehyde in PBS for 20 min and blocked in 5% goat serum in PBS with 0.1% Tween (PBT) for 20 min. For Crb staining, ovaries were fixed in 4% formaldehyde in PBS for 20 min, incubated for 2 min in 50% methanol in PBT, 2 min in 100% methanol and 2 min in 50% methanol in PBT, the washed three times for 10 min in PBT and blocked for 30 min in 10% BSA in PBT. Ovaries were incubated with primary antibodies diluted in PBT overnight at 4°C, washed four times for 5 min in PBT, incubated with secondary antibodies diluted in PBT for 3 h and washed three times for 10 min in PBT. All incubations were at room temperature unless otherwise stated. The following antibodies were used for indirect immunofluorescence: rabbit anti-PKCζ 1/600 (SAB4502380, Sigma), mouse anti-Arm 1/200 (N27-A1, Developmental Studies Hybridoma Bank), rabbit anti-Baz 1/2000 (a gift from Andreas Wodarz, University of Cologne), rat anti-Crb 1/200 (Walther et al., 2016), mouse anti-Crb 1/50 (Cq4, Developmental Studies Hybridoma Bank), guinea pig anti-Par6 1/400 (Walther et al., 2016), rat Ecad 1/20 (DCAD2, Developmental Studies Hybridoma Bank), rabbit anti-Sdt 1/400 (a gift from E. Knust), with the appropriate combination of mouse, guinea pig, rabbit and rat secondary antibodies conjugated to Dy405, Alexa488, Cy3 or Cy5 as appropriate at 1/200 each (Jackson ImmunoResearch). Samples were mounted in VectaShield with or without DAPI as appropriate and imaging was performed using a Leica SP5 or SP8 confocal microscope. Images were edited using Fiji and Adobe Photoshop 7.0.

### Quantifications

To measure pixel intensity and area of epitope staining, a threshold was applied to define the domain(s) of interest and then quantified using the wand (tracing) tool in Fiji. A minimum of 12 data points were obtained for each condition, from at least three independent retinas. To estimate relative protein distribution between the apical membrane and ZA, at least 60 ratios were measured from at least three independent retinas*.* GraphPad Prism version 7.0 for Mac was used for the statistical analyses. Data sets were tested for normality using the D'Agostino and Pearson normality test. *P* values were calculated using either the Student's *t*-test or the Mann–Whitney test in cases where the data were unpaired, or either the paired *t*-test or the Wilcoxon test in cases where the data were paired.

### FRAP

FRAP analyses in the fly retinas was performed at 40% APF as previously described (Walther et al., 2016). Live imaging was performed on a Leica SP5 confocal using a 63×1.4 NA oil immersion objective at the following settings: pixel resolution 512×512, speed 400 Hz, 15% 488 nm laser power at 20% argon laser intensity and 5× zoom. FRAP analyses of Crb::GFP (Huang et al., 2009), *par6*-Par6::GFP and GMR-Gal4; UAS-Par6 wild-type, 4A and ΔP transgenes were performed through a 5 pixel-diameter circle ROI followed by photo-bleaching with two pulses using 90% 488 nm laser power at 20% argon laser intensity. GFP recovery was recorded every 1.293 s with the previously mentioned settings for ∼300 s.

FRAP analysis in the follicular epithelium was performed as previously described (Prasad et al., 2007). Live imaging was performed on a Leica SP8 upright confocal using a 63×1.4 NA oil immersion objective at the following settings: pixel resolution 512×256, speed 400 Hz, 20% 488 nm laser power at 40% argon laser intensity and 4× zoom. FRAP analyses of Par6::GFP and Crb::GFP were performed through a 1 µm×1 µm square ROI followed by photo-bleaching with two pulses using 90% 488 nm laser power at 40% argon laser intensity. GFP recovery was recorded every 0.328 s for Par6::GFP or every 1 s for Crb::GFP with the previously mentioned settings for a ∼ 160-200 s.

For each experiment, three different *z*-axis profiles were plotted: (1) from the photo-bleached area; (2) from an equivalent area of a neighbouring non-photo-bleached photoreceptor; and (3) from an equivalent area of background. The obtained data were normalized using easyFRAP (Rapsomaniki et al., 2012) and fitted to a two-phase association curve in GraphPad Prism version 7.0 for Mac. Each data point represents the mean and error bars the s.e.m. Half-time values were determined with Prism based on the fitting curves obtained; columns represent the mean and error bars the 95% CI of each dataset. The *P* values were calculated with a two-way ANOVA test with Bonferoni's correction.

### Biochemistry

To express GST-fusion proteins, *E. coli* BL21 was transformed with the appropriate plasmids and induced with 0.2% L-arabinose or 1 mM IPTG as appropriate for 4 h at 30°C. Bacteria were lysed by sonication in 50 mM Tris HCl (pH 7.6), 50 mM NaCl, 5 mM MgCl_2_, 0.5% Triton X-100 and 10 mM DTT in the presence of protease inhibitor [EDTA-free Complete Protease Inhibitor (Roche)]. GST-fusion proteins were purified using Glutathione Sepharose 4 Fast Flow beads (GE Healthcare), washed in lysis buffer and kept on beads in lysis buffer with 1 mM DTT at 4°C. To express MBP-fusion proteins *E. coli* BL21 was transformed with the appropriate plasmids and induced with 0.3 mM IPTG for 2 h at 37°C. Bacteria were lysed in 20 mM Tris HCl (pH 7.4), 200 mM NaCl, 1 mM EDTA and 1 mM DTT. MBP-fusion proteins were purified using Amylose resin (New England Biolabs), washed in lysis buffer, eluted in lysis buffer containing 10 mM maltose, dialysed to 50 mM Tris HCl (pH 7.5), 150 mM NaCl, 5 mM MgCl_2_ and 40% glycerol, and stored at −80°C for further experiments.

*Drosophila* Schneider S2R+ cells (DGRC) were transiently transfected using Effectene Transfection Reagent (Qiagen) with empty vector *pActin-Flag* (Mock) or *pActin-par6-Flag* (WT, K23A, 4A or ΔP) and lysed in 50 mM Tris HCl (pH 7.5), 150 mM NaCl, 0.5% Triton X-100, 1 mM EDTA, protease inhibitor [EDTA-free Complete Protease Inhibitor (Roche)] and phosphatase inhibitor cocktail (Sigma). For co-immunoprecipitation experiments, Par6::Flag was immunoprecipitated with anti-FLAG M2 magnetic beads (Sigma) for 1 h at 4°C, washed three times in lysis buffer and analysed by western blot. For GST pulldown experiments, S2R+ cell lysates were added to purified GST-fusion proteins for 1 h at 4°C, washed three times in lysis buffer and analysed by western blot and Coomassie Blue staining. For *in vitro* binding assays, recombinant MBP and GST proteins were incubated for 1 h 4°C in binding buffer [50 mM Tris HCl (pH 7.5), 150 mM NaCl, 5 mM MgCl_2_, 0.5% Triton X-100] and washed three times in binding buffer. From the same sample, 20 µl was loaded onto a gel for Coomassie Blue staining and 5 µl was loaded onto a separate gel for western blot analysis.

Protein extraction was performed from eight fly heads homogenized in 30 µl of extraction buffer [125 mM NaCl, 50 mM Tris HCl (pH 7.5), 5% glycerol, 1 mM MgCl_2_, 1 mM EDTA, 0.2% NP-40, 0.5 mM DTT, protease inhibitor EDTA-free Complete Protease Inhibitor (Roche) and phosphatase inhibitor cocktail (Sigma)]. Samples were analysed by western blotting. The following antibodies were used for protein detection: anti-Flag M2 mouse 1/1000 (F3165, Sigma), anti-myc 9E10 mouse 1/1000 (sc-40, Santa-Cruz), anti-GFP (D5.1) XP rabbit 1/1000 (2956S, Cell Signaling), anti-αTubulin mouse 1/100 (AA4.3, DSHB), anti-GST rabbit 1/100,000 (G7781, Sigma) and anti-MBP mouse 1/80,000 (E8032S, New England Biolabs). Western blots were quantified using Fiji, while graphical representation and statistical analysis were performed in GraphPad Prism version 7.0 for Mac. Data are mean±s.e.m. for each dataset. *P* values were calculated with a Kruskal–Wallis test and corrected using Dunn's multiple comparison test.

## Supplementary Material

Supplementary information
