# Supplementary material for: Cdc42 defines apical identity and regulates epithelial morphogenesis by promoting apical recruitment of Par6-aPKC and Crumbs
Source: Development. 2019 Aug 12;146(15):dev175497. doi: 10.1242/dev.175497 (PMC6703713; doi:10.1242/dev.175497)
Supplement: Supplementary information [file develop-146-175497-s1.pdf]

SUPPLEMENTARY FIGURES

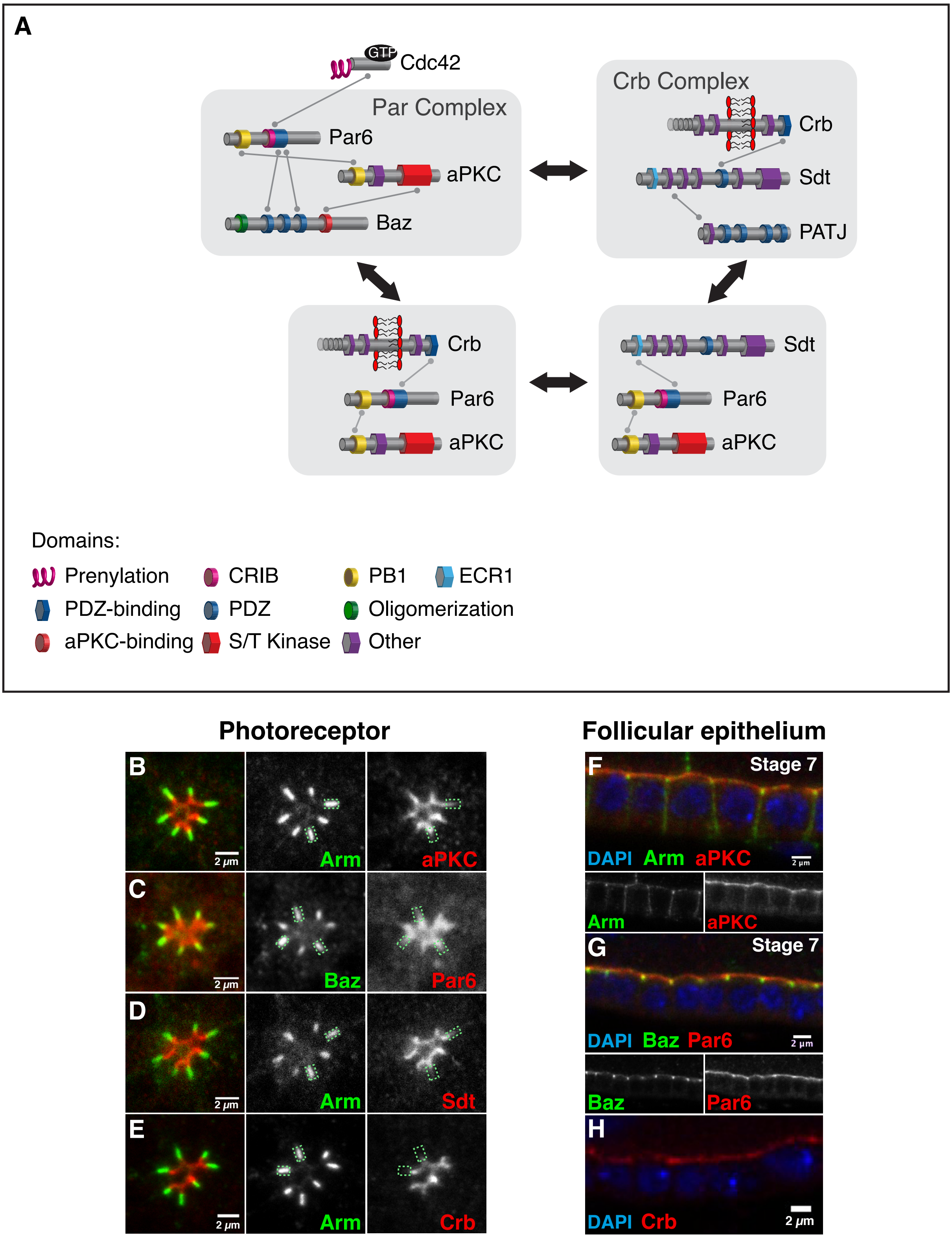

Development • Supplementary information

Figure S1: Simplified apical protein network and protein localization in the photoreceptor and follicular epithelium.

(A) Schematic representation of protein complexes that regulate epithelial polarity, and in particular the specification of the apical membrane and *ZA*. Grey lines indicate that these respective protein domains can interact with and bind to each other *in vitro*. (**B-E**) Photoreceptors at 40% after puparium formation stained for (**B**) Arm (green) and aPKC (red), (**C**) Baz (green) and Par6 (red), (**D**) Arm (green) and Sdt (red), and (**E**) Arm (green) and Crb (red). Green rectangles delineate the *ZA* to show the relative overlap between Arm/Baz and aPKC/Par6/Sdt/Crb. (**F-H**) Wild-type cuboidal follicular epithelial cells from stage 7 follicles stained for (**F**) DAPI (blue), Arm (green) and aPKC (red), (**G**) DAPI (blue), Baz (green) and Par6 (red), and (**H**) DAPI (blue) and Crb (red).

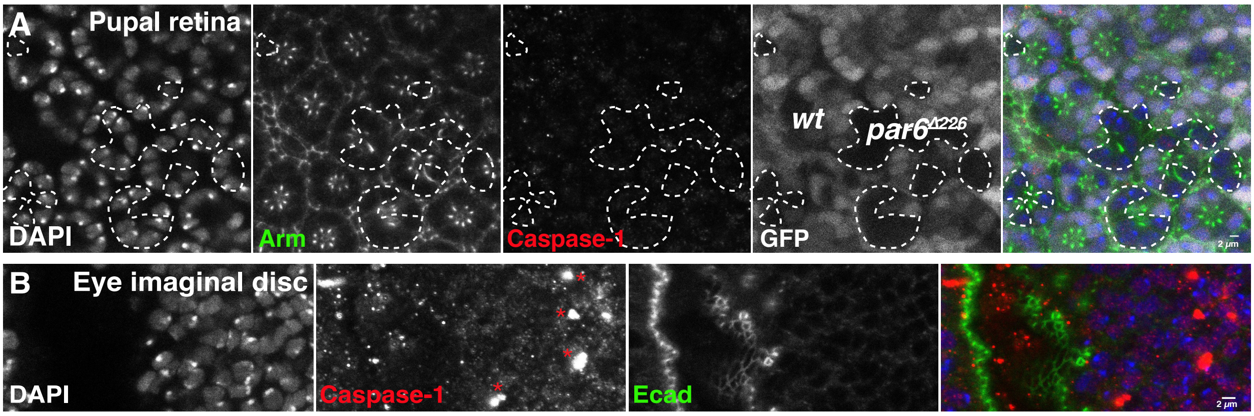

Figure S2: *par6* mutant pupal photoreceptors do not undergo apoptosis

(A) Caspase-1 staining (red) of a representative *par6*$^{\Delta 226}$ mosaic pupal retina. DAPI marks the nuclei (grey; blue in merged panel), Arm (green). *par6*$^{\Delta 226}$ mutant cells are circled using dashed lines. **(B)** Control for Caspase-1 staining using eye imaginal discs. DAPI marks the nuclei (grey; blue in merged panel), Ecad (green) and Caspase-1 (red). Apoptotic cells are indicted with red asterisks (*).

Development • Supplementary information

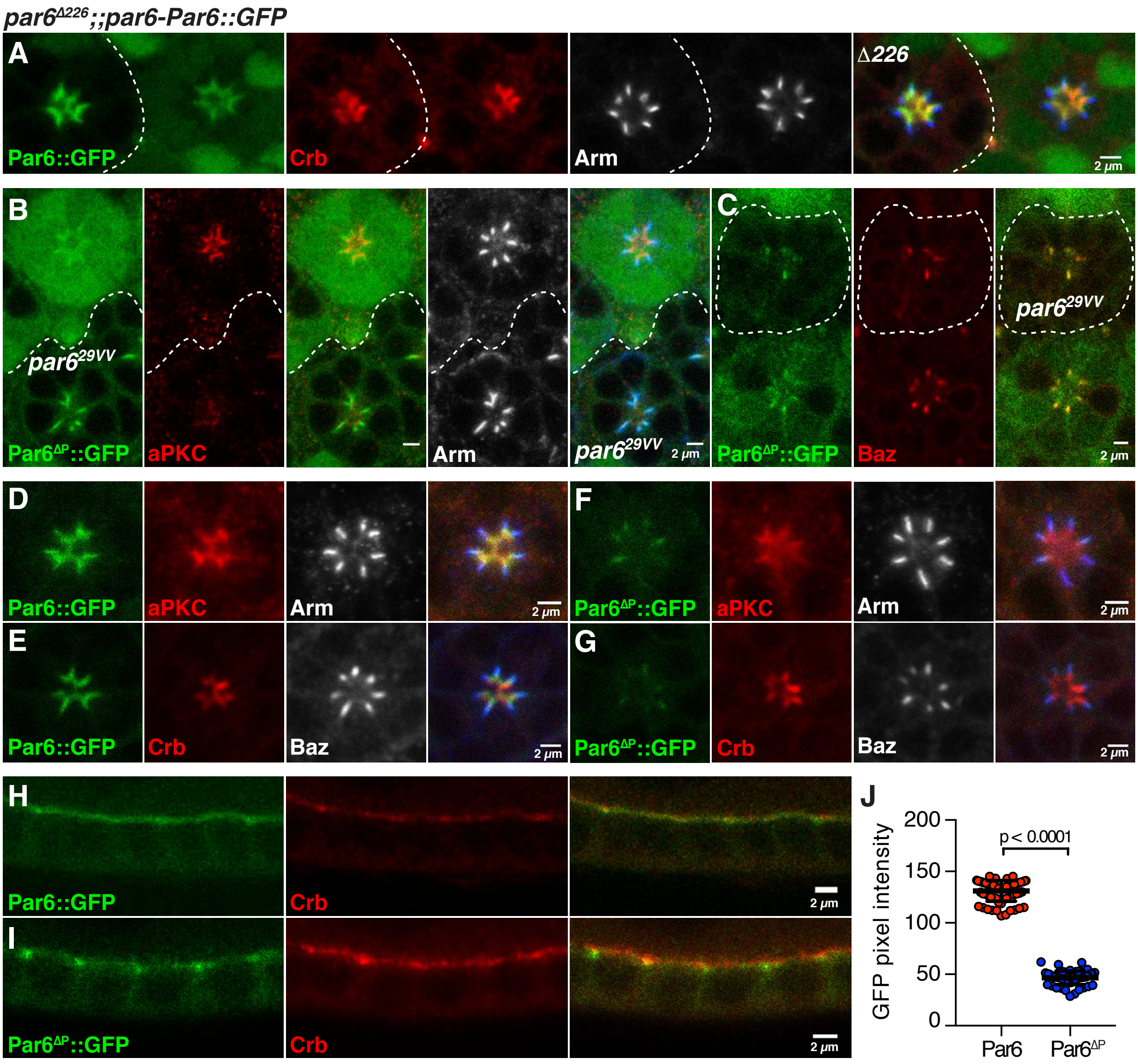

Figure S3: Par6 binding to Cdc42 promotes the apical localization of Par6-aPKC

(A) *par6*$^{\Delta 226}$ pupal retinal clones expressing *par6-Par6::GFP* (green). *par6* $^{\Delta 226}$ mutant cells labeled by loss of nuclear GFP signal (green) and stained for Crb (red) and Arm (grey). **(B - C)** *par6*^{29VV} pupal retinal clones expressing *par6-Par6*$^{\Delta P}$*::GFP* (green). *par62*^{29VV} mutant cells are labeled by loss of cytosolic GFP signal and stained for **(B)**

Development • Supplementary information

aPKC (red) and Arm (grey) and **(C)** Baz (red). **(D-E)** Wild-type pupal photoreceptors expressing *par6-Par6::GFP* (green) and stained for **(D)** aPKC (red) and Arm (grey); and **(E)** Crb (red) and Baz (grey). **(F-G)** Wild-type pupal photoreceptors expressing *par6-Par6*$^{\Delta P}$*::GFP* imaged at the same confocal settings as **(D-E)** and stained for **(F)** aPKC (red) and Arm (grey); and **(G)** Crb (red) and Baz (grey). **(H-I)** Follicular epithelial cells expressing **(H)** *par6-Par6::GFP* (green) or **(I)** *par6-Par6*$^{\Delta P}$*::GFP* (green) and stained for Crb (red). **(J)** Quantification of the mean intensity of *par6-Par6::GFP and par6-Par6* $^{\Delta P}$*::GFP.* For each quantification, at least 63 measurements were taken from 5 retinas. In all figures, where appropriate, the grey channel is shown in blue in the merge. Scale bars = 2 microns.

Development • Supplementary information

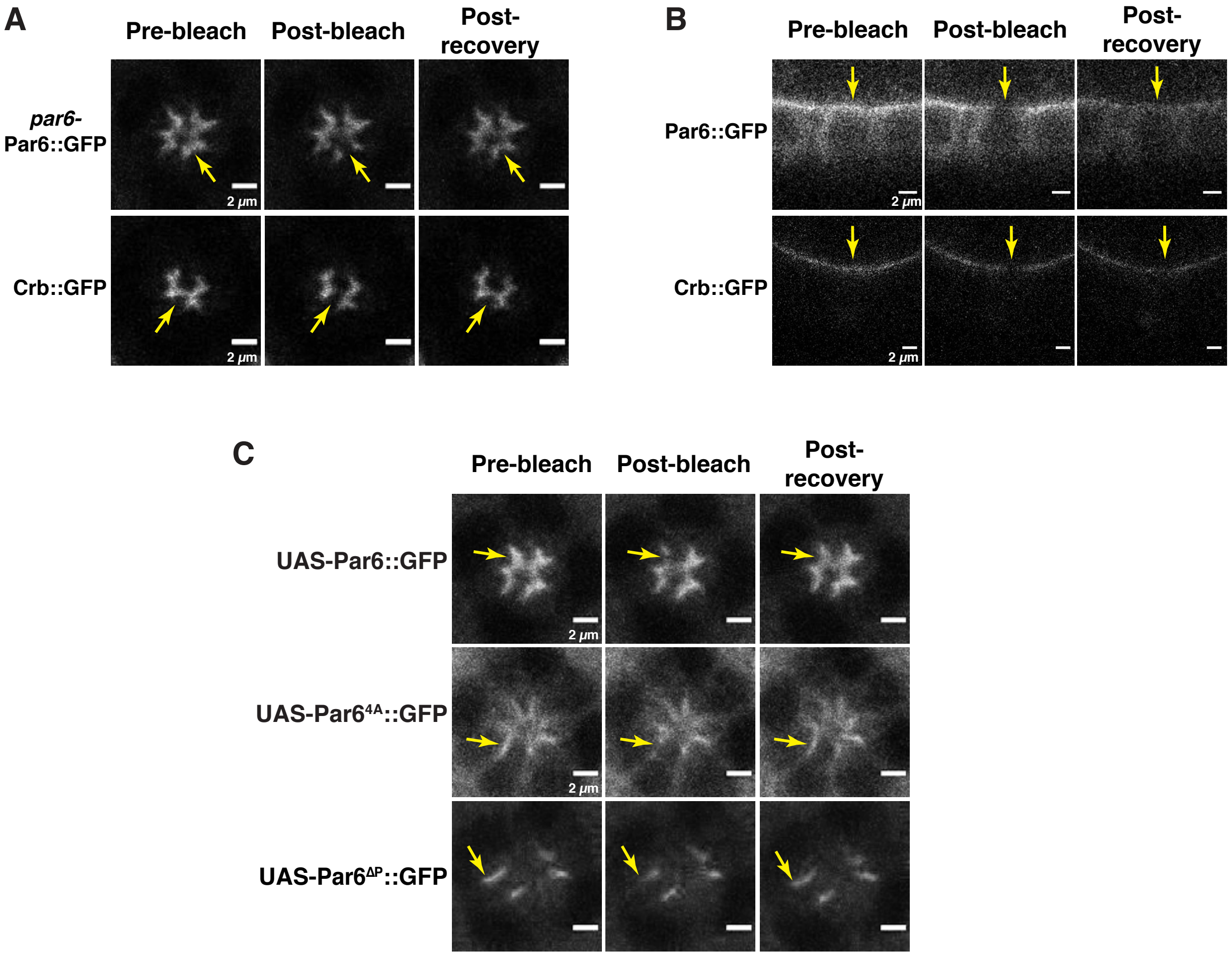

Figure S4: Crb promotes apical retention of Par6

(**A-C**) Still images taken from representative time lapses of FRAP experiments. In all panels, the photobleached area is indicated with a yellow arrow. (**A**) Still images of *par6*-Par6::GFP and Crb::GFP FRAP, performed in pupal photoreceptors. (**B**) Still images of *par6*-Par6::GFP and Crb::GFP FRAP, performed in follicular epithelia. (**C**) Still images of UAS-Par6::GFP FRAP, UAS-Par6^{4A}::GFP and UAS-Par6$^{\Delta P}$::GFP FRAP, performed in pupal photoreceptors. All scale bars = 2 microns.
